# An interlaboratory comparison of 16S rRNA gene-based terminal restriction fragment length polymorphism and sequencing methods for assessing microbial diversity of seafloor basalts

**DOI:** 10.1111/j.1462-2920.2009.01899.x

**Published:** 2009-07

**Authors:** Beth Orcutt, Brad Bailey, Hubert Staudigel, Bradley M Tebo, Katrina J Edwards

**Affiliations:** 1 Geomicrobiology Group, Department of Biological Sciences, Marine Environmental Biology, University of Southern CaliforniaLos Angeles, CA 90089, USA; 2 Institute of Geophysics and Planetary Physics, Scripps Institution of Oceanography, University of CaliforniaLa Jolla, CA 92037, USA; 3 Division of Environmental and Biomolecular Systems, Oregon Health and Science UniversityBeaverton, OR 97006, USA

## Abstract

We present an interlaboratory comparison between full-length 16S rRNA gene sequence analysis and terminal restriction fragment length polymorphism (TRFLP) for microbial communities hosted on seafloor basaltic lavas, with the goal of evaluating how similarly these two different DNA-based methods used in two independent labs would estimate the microbial diversity of the same basalt samples. Two samples were selected for these analyses based on differences detected in the overall levels of microbial diversity between them. Richness estimators indicate that TRFLP analysis significantly underestimates the richness of the relatively high-diversity seafloor basalt microbial community: at least 50% of species from the high-diversity site are missed by TRFLP. However, both methods reveal similar dominant species from the samples, and they predict similar levels of relative diversity between the two samples. Importantly, these results suggest that DNA-extraction or PCR-related bias between the two laboratories is minimal. We conclude that TRFLP may be useful for relative comparisons of diversity between basalt samples, for identifying dominant species, and for estimating the richness and evenness of low-diversity, skewed populations of seafloor basalt microbial communities, but that TRFLP may miss a majority of species in relatively highly diverse samples.

## Introduction

Application of the proper metrics for assessing, measuring and quantifying microbial populations in complex natural systems is a current and long-standing challenge in microbial diversity studies (reviewed in Hughes *et al*., 2001). Community genetic fingerprinting methods, such as terminal restriction fragment length polymorphism (TRFLP; Liu *et al*., 1997), have been used repeatedly to investigate comparative community composition owing to the procedures being relatively rapid, high-throughput and inexpensive. For highly diverse microbial communities, however, there are conflicting results whether different fingerprinting methods determine similar (e.g. Hartmann *et al*., 2005) or different (e.g. Danovaro *et al*., 2006) levels of diversity, although part of the disagreement between methodological approaches may be due to focusing in some cases on the coding 16S rRNA gene and in others on the more highly variable, non-coding intergenic regions of the DNA sequence. In addition, the utility of fingerprinting methods to accurately calculate community diversity in complex communities has been criticized due to inability of the methods to detect low-abundance (< 1% of the community) and rare taxa (Dunbar *et al*., 2000; Blackwood *et al*., 2003; Engebretson and Moyer, 2003; Bent *et al*., 2007). Recent innovations, such as pyrosequencing, now suggest that microbial communities contain even greater diversity than previously accepted (Sogin *et al*., 2006; Huber *et al*., 2007). It is unknown how the rare members of any ecosystem translate to ecological or biogeochemical function, although it is suggested that the rare taxa may serve as a reservoir of genomic innovation to allow microbial communities to adapt to changing environmental conditions (Sogin *et al*., 2006). If fingerprinting methods exclude rare taxa, are they useful for predicting metabolic potential in a microbial ecosystem?

A further challenge for microbial ecologists is the reproducibility of microbial biodiversity surveys on similar samples using different methods. As one example, surveys conducted on similar deep marine subsurface sediments using different methods have resulted in contradictory interpretations of whether the domain *Bacteria* or *Archaea* dominate the deep biosphere microbial community (Biddle *et al*., 2006; Inagaki *et al*., 2006; Schippers and Neretin, 2006; Sørensen and Teske, 2006; Lipp *et al*., 2008). In order to assess global and local biodiversity patterns and to correctly correlate microbial community form with function, it is essential that methods provide predictable and ecologically meaningful information, even if biased (Hughes Martiny *et al*., 2006).

In the present study, two independent laboratories utilized different approaches that target the 16S rRNA gene to evaluate diversity of seafloor basalt samples, and the results were cross-compared for the purposes of evaluating methods and interlaboratory biases in biodiversity assessments. Previous studies have shown that seafloor basalts harbour some of the most diverse microbial communities on Earth (Santelli *et al*., 2008). Here, a set of two seafloor basalt samples collected from the Lô'ihi Seamount were analysed independently by both TRFLP analysis and 16S rRNA gene clone libraries as well as *in silico* TRFLP analysis of the 16S rRNA sequences. Commonly used diversity indices were applied to the results of both the community fingerprinting analysis by TRFLP as well as to the similarity matrices calculated from the gene sequences. As compared with previous computer-simulated comparisons between TRFLP patterns and 16S rRNA gene sequences (Liu *et al*., 1997; Engebretson and Moyer, 2003; Blackwood *et al*., 2007) and comparisons between restriction digests of 16S rRNA gene clones amplified from soils (Dunbar *et al*., 1999; 2000), this study evaluated empirically derived diversity assessments generated independently by two labs from the same samples.

## Results

Two seafloor basalt samples were the focus of this comparative study, which had recently been identified as representing relatively high- and low-diversity end members among a suite of basalt samples from Hawai'i (Santelli *et al*., 2008). The ‘high diversity’ sample originated from the Pisces Peak location on the Lô'ihi Seamount off the south coast of the big island of Hawai'i (sample PV549X2). The ‘low diversity’ end member was collected from the South Rift location on the southern flank of Lô'ihi (sample PV547X3).

**Fig. 1:**
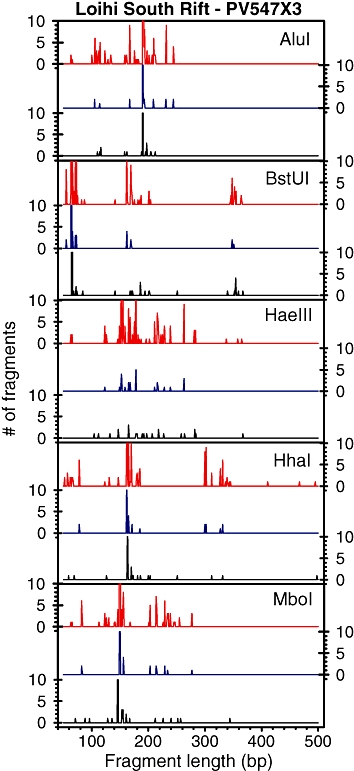
Comparison of TRFLP patterns from the South Rift sample. Red lines indicate number of fragments from the measured TRFLP with the respective enzymes when assuming a threshold cut-off of 15 fluorescence units; blue lines indicate the number of fragments from the measured TRFLP with the respective enzymes when assuming a threshold cut-off of 50 fluorescence units; and black lines indicate the number of TRFLP fragments generated from *in silico* digest of nearly full-length 16S rRNA sequences using the respective enzyme pre-sets in the tRF-cut program in ARB. Some peaks are larger than *y*-scale given, small *y*-scales given to highlight rare OTUs (shorter peaks).

**Fig. 2:**
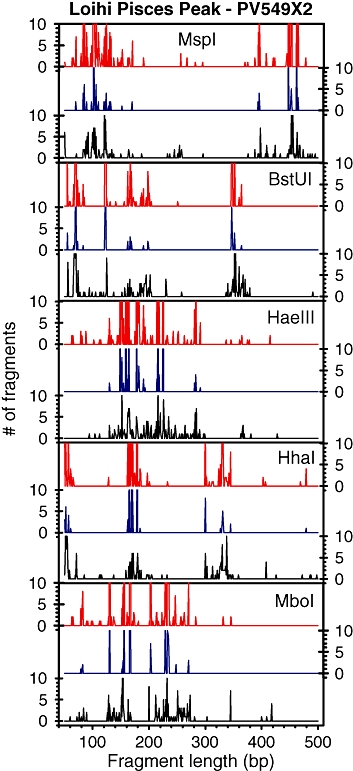
Comparison of TRFLP patterns from the Pisces Peak sample. Red lines indicate number of fragments from the measured TRFLP with the respective enzymes when assuming a threshold cut-off of 15 fluorescence units; blue lines indicate the number of fragments from the measured TRFLP with the respective enzymes when assuming a threshold cut-off of 50 fluorescence units; and black lines indicate the number of TRFLP fragments generated from *in silico* digest of nearly full-length 16S rRNA sequences using the respective enzyme pre-sets in the tRF-cut program in ARB. Some peaks are larger than *y*-scale given, small *y*-scales given to highlight rare OTUs (shorter peaks).

From two subsamples of the same rocks, 16S rRNA gene clone libraries were constructed following DNA extraction in one lab, while TRFLP analysis was performed with DNA extracted in another lab. The two labs used different DNA extraction protocols and slightly different primers for PCR amplification (see *Experimental procedures* below). From the clone library data, which contained 246 sequences from the high-diversity sample and 71 sequences from the low-diversity sample, the microbial diversity and richness were estimated from sequence alignments using the program dotur (Schloss and Handelsman, 2005) at a ≥ 97% sequence similarity definition for species, as described elsewhere (Santelli *et al*., 2008). To allow comparison of the clone library data with the measured TRFLP analyses (see below), an *in silico* terminal restriction digestion (Ricke *et al*., 2005) was performed on the clone library sequences to generate theoretical fragment patterns that could be directly compared with the measured TRFLP patterns. Figures 1 and 2 present the measured and predicted TRFLP patterns from the South Rift and Pisces Peak samples respectively.

To evaluate how each method predicted microbial community diversity, a suite of classical non-parametric richness and evenness indices were calculated from the observed operational taxonomic unit (OTU) distribution generated by each of the sampling methods (Table 1). As shown previously (Santelli *et al*., 2008), diversity and richness estimates based on clone library sequence alignments via dotur indicated that the Pisces Peak sample had higher richness with roughly 400 species (from abundance-based coverage (ACE) species richness estimator and Chao1 species richness estimator) as compared with the South Rift basalt, which was estimated to support approximately 100 species (from ACE and Chao1). Calculation of the Shannon–Weaver index (*H′*) and the Simpson index (*D*) from the sequence alignments also indicated that the Pisces Peak sample had a higher diversity than the South Rift basalt (respectively, diversity index of 4.77 versus 1.83 for *H′* and 0.0071 versus 0.38 for *D*). In addition, the Shannon evenness index (*J′*) indicated that the Pisces Peak sample had a more even microbial population (i.e. index closer to 1) than the South Rift sample.

**Table 1:** Diversity and evenness indices calculated from various methods for the two seafloor basalt samples considered in this study.

Sample	Measure	ACE^a^	Chao^b^	Shannon *H*′^c^	Shannon *J*′^d^	Simpson^e^	*S*_obs_^f^	n_1_^g^	n_2_^h^
Pisces Peak	dotur^i^	404	416	4.77	0.95	0.0071	148	104	19
	*is*TRFLP^j^	169 ± 26	168 ± 25	4.16 ± 0.2	0.91 ± 0.02	0.020 ± 0.08	96 ± 11	50 ± 8	17 ± 4
	TRFLP_15_^k^	76 ± 14	69 ± 10	3.08 ± 0.2	0.76 ± 0.04	0.08 ± 0.02	59 ± 10	17 ± 4	15 ± 6
	TRFLP_50_^l^	25 ± 2	23 ± 2	2.55 ± 0.2	0.84 ± 0.04	0.10 ± 0.03	21 ± 3	4 ± 1	4 ± 4
South Rift	dotur^i^	110	87.3	1.83	0.58	0.38	24	20	2
	*is*TRFLP^j^	86 ± 19	91 ± 31	1.92 ± 0.2	0.62 ± 0.05	0.32 ± 0.08	22 ± 2	17 ± 2	2 ± 1
	TRFLP_15_^k^	47 ± 8	43 ± 7	2.50 ± 0.5	0.70 ± 0.13	0.17 ± 0.11	35 ± 4	11 ± 3	9 ± 2
	TRFLP_50_^l^	13 ± 4	13 ± 4	1.81 ± 0.4	0.75 ± 0.13	0.26 ± 0.15	11 ± 2	4 ± 2	4 ± 1

a ACE, predicted species richness using abundance-based coverage estimator, calculated using formulation from Chao and Lee (1992).

b Chao, predicted species richness using Chao1 estimator, calculated using formulation from Chao and colleagues (1992).

c Shannon H′, Shannon–Weaver index of diversity, higher numbers indicate higher diversity, calculated using formulation from Schloss and Handelsman (2005).

d Shannon J′, Shannon evenness, values can range from 0 to 1, with 1 representing perfect evenness, calculated using formulation from Schloss and Handelsman (2005).

e Simpson, Simpson index of diversity, values can range from 0 to 1 with higher values indicating a skewed population with dominant members, calculated using formulation from Schloss and Handelsman (2005).

f *S*_obs_, observed OTUs.

g Number of OTUs with only one member.

h Number of OTUs with only two members.

i dotur, based on neighbor-joining alignment at 97% sequence similarity of nearly full-length 16S rRNA sequences in ARB following application of a filter for bacteria for the positions 1218–42590 and the Jukes Cantor correction.

j *is*TRFLP: estimated using all of the TRFLP fragments generated from the *in silico* digest of the sequences recovered from 16S rRNA clone library; average of five digests with different restriction enzymes ± standard deviation of the average.

k TRFLP_15_: estimated from the actual TRFLP fragments assuming a threshold cut-off value of 15 fluorescence units; average of five digests with different restriction enzymes ± standard deviation of the average.

l TRFLP_50_: estimated from the actual TRFLP fragments assuming a threshold cut-off value of 50 fluorescence units; average of five digests with different restriction enzymes ± standard deviation of the average.

When calculating species richness from the predicted *in silico* TRFLP OTU distributions (*is*TRFLP, Table 1), the ACE and Chao1 estimates (86 ± 19 and 91 ± 31, respectively, with the variation attributable to the differences between restriction enzyme considered) were similar to the sequence-derived values (roughly 100) for the low-diversity sample from South Rift, but the predicted species richness for the high-diversity sample from Pisces Peak was significantly lower, with a predicted richness of roughly 170 species as compared with ∼400. Additionally, the Shannon *H′* and Simpson richness indices estimated lower richness from the *in silico* TRFLP OTU distributions for the Pisces Peak sample, but similar values were calculated for the South Rift sample by both methods. Notably, the calculation of species evenness (*J′*) was similar by either method for both samples.

The prediction of species richness from the ACE and Chao1 estimators from the measured TRFLP analysis was significantly lower than from the other methods for both samples, regardless of whether a threshold cut-off value of 15 or 50 fluorescence units was assumed for converting TRFLP electropherogram data into species abundance patterns (see *Experimental procedures* section). For example, there were a predicted 76 ± 14 species in the Pisces Peak basalt and 47 ± 8 species in the South Rift basalt when assuming a 15 fluorescence unit cut-off value (TRFLP_15_, Table 1); even lower estimates of 25 ± 2 species in the Pisces Peak sample and 13 ± 4 species in the South Rift sample were calculated when assuming a threshold value of 50 fluorescence units (TRFLP_50_, Table 1). The Shannon and Simpson richness indices also estimated significantly lower diversity for the Pisces Peak sample from the measured TRFLP patterns than from the other methods; however, the species evenness prediction (*J′*) was similar but slightly lower. In contrast, for the lower diversity South Rift sample, the Shannon and Simpson richness indices estimated from the measured TRFLP data predicted a similar or slightly more diverse community than estimated using the other sequence based and *is*TRFLP methods. Although the OTU sample sizes (*S*_obs_, Table 1) for the low-diversity sample are relatively small, they are still expected to be significant enough that the ACE and Chao1 richness estimators are not overly biased by sample size [i.e. while some sample size bias on the predicted richness estimators is expected (see Youssef and Elshahed, 2008), *S*_obs_ in our samples is greater than the square root of twice the estimate of richness; Colwell and Coddington, 1994]. Notably, all methods calculate that the Pisces Peak sample harbours a more diverse microbial community than the South Rift sample.

A peak-by-peak correlation between the measured TRFLP fragment peaks and those predicted from the *in silico* digestion of the clone library sequences (Figs 1 and 2, Table 2) reveals that peaks predicted from the *in silico* TRFLP digestion were correlated perfectly or within 1–2 base pairs (bp) to 37% and 72% of the peaks from the measured TRFLP_15_ patterns for the South Rift and Pisces Peak samples respectively (the numbers shift to 45% and 88% of peaks respectively, when using TRFLP_50_). The viability of correlating peaks offset by 1–2 bp is validated by the variation between true and observed fragments based on the relative composition of the DNA sequences (Kaplan and Kitts, 2003). When weighted by frequency of appearance of a particular fragment, this corresponds to 67% and 92%, respectively, of PCR amplicons from the measured TRFLP_15_ analysis being matched by fragments in the *in silico* TRFLP analysis (or 75% and 93% when using TRFLP_50_). While the dominant species were recovered in both analyses, a larger proportion of rare species was recovered in the clone library data. Roughly one-third to two-thirds of the TRFLP peaks predicted from the *in silico* digestion are not matched in the measured TRFLP_15_ or TRFLP_50_ analyses.

**Table 2:** Peak-to-peak correlation (as a percentage of total OTUs) between measured TRFLP patterns and predicted TRFLP patterns from an *in silico* digestion of 16S rRNA clone library data.

	South Rift		Pisces Peak	
	15 f.u. cut-off	50 f.u. cut-off	15 f.u. cut-off	50 f.u. cut-off
TRFLP peaks perfect match	12 ± 4	8 ± 10	42 ± 4	56 ± 11
TRFLP peaks off by 1–2 bp	25 ± 13	37 ± 29	30 ± 4	32 ± 12
TRFLP peaks not matched	63 ± 12	55 ± 20	28 ± 3	12 ± 7
TRFLP ‘members’ identified	67 ± 11	75 ± 9	92 ± 3	93 ± 3
*in silico* peaks perfect match	24 ± 13	6 ± 8	29 ± 4	15 ± 4
*in silico* peaks off by 1–2 bp	45 ± 15	20 ± 15	21 ± 4	8 ± 3
*in silico* peaks not matched	30 ± 7	77 ± 3	50 ± 6	77 ± 3

Data are presented for the correlations when the measured TRFLP fragments are assumed versus both 15- and 50-fluorescence-unit (f.u.) threshold cut-off values.

## Discussion

Seafloor basalts harbour some of the most diverse microbial communities on Earth (Santelli *et al*., 2008). Previous analysis indicates that the two samples used in this study represent both relatively low (Lô'ihi South Rift basalt) and high (Lô'ihi Pisces Peak basalt) diversity end members (Santelli *et al*., 2008). A major goal of the present study was to evaluate how similarly two different DNA-based methods (i.e. TRFLP and 16S rRNA gene clone libraries) conducted in two independent labs would estimate the microbial diversity of these basalt samples. Although there are differences between the two methodological approaches to examining diversity of seafloor basalts (i.e. different DNA extraction methods and PCR conditions, see *Experimental procedures* below), it is notable that both methods retrieved similar dominant species from the samples, and that they predicted similar levels of relative diversity between the two samples. For instance, as shown in Fig. 1, the major species (i.e. largest peak) that dominated the low-diversity South Rift sample was retrieved by both methods, regardless of the enzyme considered for TRFLP. Similarly, the measured and *in silico* TRFLP patterns from the Pisces Peak sample are qualitatively similar, with dominant peaks often matching between the two methods (Fig. 2). Although predicting a much lower richness level, the calculation of richness from the measured TRFLP analyses also indicates that the Pisces Peak basalt microbial community is more diverse than the South Rift basalt community (Table 1). Importantly, these results suggest that there was minimal apparent DNA-extraction or PCR-related bias between the two laboratories although different protocols were used. Although, theoretically, it is not unexpected that the two approaches would uncover similar communities, this is rarely evaluated in molecular diversity studies and is often a considerable uncertainty in making cross-comparisons between studies.

The comparison of richness estimators calculated from the two data sets reveals that TRFLP analysis significantly underestimated the richness of the relatively high-diversity seafloor basalt microbial community (Table 1). Depending on the richness estimator considered, richness predicted by TRFLP analysis for the low-diversity sample was roughly the same (using Shannon and Simpson indices) or lower (ACE and Chao1 estimators) than the richness predicted by sequence similarity. Although the richness estimates were different between the two methodologies, the predictions of community evenness were similar. It should be emphasized that the diversity indices calculated based on 16S rRNA gene clone similarity used a definition of ≥ 97% similar sequence for determining the number of species. If a higher threshold had been used (i.e. 99% sequence similarity), the number of species predicted from the clone library data would have been even greater. The ≥ 97% cut-off is a commonly recognized level for comparative analysis in environmental microbial communities. Nonetheless, it is likely that at least 50% of species from the Pisces Peak samples are missed by the laboratory-based TRFLP analysis (Table 2, assuming no bias from DNA extraction of PCR primer conditions), as parametric-based rarefaction analysis of the sequence similarity data for this sample (from Santelli *et al*., 2008), which is a measure of the number of species observed per sampling effort, indicates that significantly more sampling would be needed to measure the full diversity of the sample.

There are at least two reasons why the richness estimated by TRFLP is lower than that estimated from clone library sequence alignment, as suggested previously for simulated comparisons of TRFLP and clone sequences (Engebretson and Moyer, 2003; Blackwood *et al*., 2007). The first explanation is that TRFLP misses the majority of rare species, likely due to detection limits for resolving unique sequence variants. For example, if the 16S rRNA gene from a rare species did not generate enough fluorescently labelled PCR amplicons, this species may be missed by the limit of detection for the TRFLP analysis. An evaluation of how many rare species are captured by each method can be seen in the number of species that were observed only once (n_1_) or twice (n_2_) using the various methods (Table 1). For instance, in the clone libraries, 104 and 20 different species were observed only once in the Pisces Peak and South Rift samples, respectively, whereas only 17 and 11 species, respectively, were observed only once in the measured TRFLP_15_ analysis, and only four species were observed once in each sample in the TRFLP_50_ analysis. This trend is also evident in Figs 1 and 2, where more small peaks appear in the *in silico* TRFLP patterns than in the measured TRFLP_15_ or TRFLP_50_ patterns.

The second explanation for the lower estimations of richness from TRFLP as compared with the clone library data is the occurrence of binning in TRFLP, where two different species are counted as one TRFLP OTU because they generate the same size fragment. This can be shown empirically in the *in silico* TRFLP analysis of the clone library data, especially for the higher diversity Pisces Peak sample. For one example, the peak seen in the *in silico BstUI* digestion of the Pisces Peak sample at a fragment size of 70 bp (Fig. 2) contains predicted fragments derived from a diverse range of species from the phyla *Bacteriodetes*, *Firmicutes* and *Planctomycetes* as well as the Alpha-, Delta-, and Gamma- divisions of the phyla *Proteobacteria (*data not shown). *BstUI* digestion generates 70 bp fragments from 14 different species as defined by gene sequence similarity, which in the predicted TRFLP analysis would be binned as one peak, or species. This result of multiple species binning into one OTU has been demonstrated before (e.g. Clement *et al*., 1998). In the high-diversity sample from Pisces Peak, the degree of binning is evident in the difference between the richness estimators calculated from the sequence alignment and the *in silico* digestions (dotur versus *is*TRFLP in Table 1) – the *in silico*-based calculations predict nearly two-thirds less richness when estimated by the ACE and Chao1 estimators. For comparison, in randomly simulated microbial communities with richness levels of 100 species, the restriction enzymes considered in this study would correctly assign 60% or less of the expected OTUs (Engebretson and Moyer, 2003). Both of the above trends of binning and exclusion of rare taxa are also evident in a comparison of diversity indices calculated by TRFLP and restriction digestion of 16S rRNA gene clones in soils (Dunbar *et al*., 1999; 2000). Binning is evident regardless of which restriction enzyme is considered. In principal, though, it is possible to resolve specific instances of binning in individual data sets by employing multiple restriction enzymes, as sequences that bin together with one restriction enzyme might produce different-sized fragments when targeted by a different restriction enzyme.

Results of these studies indicate that TRFLP could be useful for relative comparisons of diversity between samples, for identifying dominant species and for estimating the richness and evenness of low-diversity, skewed populations of seafloor basalt microbial communities. This observation supports the claim by Engebretson and Moyer (2003) that TRFLP is a robust technique for identifying dominant populations and for calculating diversity statistics in low-diversity communities. Furthermore, these findings verify that TRFLP will miss the majority of low-abundance taxa in highly diverse communities.

## Experimental procedures

### Sample collection

As described previously (Santelli *et al*., 2008) the basaltic lava samples for this study were collected from basalt outcrops around the big island of Hawai'i during cruise KOK 02-24 on R/V *Ka'imikai-o-Kanaloa* in November 2002 using the manned *Pisces V* submersible. Rock samples were placed in bioboxes containing distilled freshwater, allowed to fill with ambient bottom seawater, sealed to minimize contamination from the upper water column, and brought to the surface for immediate processing. Upon retrieval shipboard, basalt samples were handled aseptically using flame- and/or ethanol-sterilized equipment. One portion of the sample was stored at −80°C in a sterile Whirlpak bag for 16S rRNA clone library construction at the Woods Hole Oceanographic Institute; the other portion was stored in sterile cryovials at −80°C for TRFLP analysis at the Scripps Institution of Oceanography.

### TRFLP analysis

DNA was extracted from samples for TRFLP analysis using the QBioGene fastdna spin kit for Soil (http://www.QBioGene.com, Catalogue #6560-200) and purifying/desalting resultant DNA with a microcon centrifugal filter device (http://www.millipore.com, Cat# 42416). The 16S rRNA gene was amplified via polymerase chain reaction (PCR) using universal PCR primers with an annealing temperature of 56°C. The forward primer 68F (5-TNA NAC ATG CAA GTC GRR CG) was fluorescently labelled on the 5′ end with 6-FAM (6-carboxyfluorescein); the reverse primer was 1492R (5-RGY TAC CTT GTT ACG ACT T). PCR amplification products were visualized and assayed for size by 1% gel electrophoresis against a 1 kb ladder DNA standard. Fluorescently labelled PCR products were then digested for 6–8 h with various restriction enzymes: AluI, HaeIII, HhaI, MboI, MspI and RsaI (New England Biolabs, Beverly, Mass.). The end-labelled SSU rDNA fragments were separated by polyacrylamide gel electrophoresis against the Genescan-500 ROX size standard with an ABI model 377 automated DNA sequencer, and the data were analysed with the Genescan software (Applied Biosystems, Foster City, Calif.). Electrophoretic resolution of the TRFLP fragments ranged from 50 to 500 bp in length. For the application of richness estimators, it was assumed that one PCR amplicon corresponded to the threshold detection limits of the TRFLP analysis; all peaks were then divided by this value to calculate the frequency or abundance of the OTUs. Two threshold detection limits were evaluated: a peak height of 15 fluorescence units, which corresponds most closely to the signal-to-noise ratio observed in the TRFLP analyses in this study, as well as a higher cut-off criteria of 50 fluorescence units, which is more commonly used in the literature (e.g. Danovaro *et al*., 2006; Culman *et al*., 2008).

### 16S rRNA gene clone library construction and *in silico* TRFLP analysis

Procedures for creating 16S rRNA gene clone libraries from the two samples have been presented previously (Santelli *et al*., 2008). Briefly, DNA was extracted from the samples using the Ultraclean soil DNA kit (MoBio Laboratories) following a slightly modified manufacturer's protocol. Prior to bead beating, the mixture was incubated for 10 min at 70°C after which 200 μg of polyadenylic acid (poly A) was added. The 16S rRNA gene was amplified using the bacteria-specific primer 8F (5′-AGA GTT TGA TCC TGG CTC AG-3′) and universal primer 1492R (5′-GGT TAC CTT GTT ACG ACT T-3′). The sequences used in this study (from Santelli *et al*., 2008) are deposited in GenBank under the Accession Numbers EU491020-EU491090 (from Lô'ihi South Rift) and EU491091-EU491336 (from Lô'ihi Pisces Peak). The computer program dotur (Schloss and Handelsman, 2005) was used to calculate species richness estimators from genetic sequence distances using a 97% sequence similarity definition. Using the add-on program tRF-cut (Ricke *et al*., 2005) for ARB (Ludwig *et al*., 2004), an *in silico* TRFLP digestion of the nearly full-length 16S rRNA sequences was performed using the restriction enzyme pre-sets in the program for *AluI*, *HaeIII*, *HhaI*, *MboI*, *MspI* and *RsaI* assuming that the 68F primer as above was the labelled primer. The predicted fragments were then grouped by base-pair length to generate abundance curves replicating a TRFLP pattern. Fragments of the same size were grouped into one OTU; the frequency of each OTU was assumed from the number of sequences in each OTU.

### Calculation of diversity indices

The number and abundance of OTUs calculated in the various data sets were used to evaluate classical non-parametric richness and evenness indices, including the ACE estimator (Chao and Lee, 1992), the Chao1 estimator (Chao, 1984), the Shannon–Weaver index of diversity (*H′*), the Shannon evenness index (*J′*) and the Simpson index of diversity (*D*). *H′*, *J′* and *D* were calculated as formulated in Schloss and Handelsman (2005).

## References

[b1] Bent SJ, Pierson JD, Forney LJ (2007). Measuring species richness based on microbial community fingerprints: the Emperor has no clothes. Appl Environ Microbiol.

[b2] Biddle JF, Lipp JS, Lever M, Lloyd KG, Sørensen KB, Anderson K (2006). Heterotrophic Archaea dominate sedimentary subsurface ecosystems off Peru. Pro Natl Acad Sci U S A.

[b3] Blackwood CB, Marsh T, Kim SH, Paul EA (2003). Terminal restriction fragment length polymorphism data analysis for quantitative comprison of microbial communities. Appl Environ Microbiol.

[b4] Blackwood CB, Hudleston D, Zak DR, Buyer JS (2007). Interpreting ecological diversity indices applied to terminal restriction fragment length polymorphism data: insights from simulated microbial communities. Appl Environ Microbiol.

[b5] Chao A (1984). Non-parametric estimation of the number of classes in a population. Scand J Stat.

[b6] Chao A, Lee SM (1992). Estimating the number of classes via sample coverage. J Am Stat Assoc.

[b7] Clement BG, Kehl LE, Debord KL, Kitts CL (1998). Terminal restriction fragment patterns (TRFPs), a rapid, PCR-based method for the comparison of complex bacterial communities. J Microbiol Methods.

[b8] Colwell RK, Coddington JA (1994). Estimating terrestrial biodiversity through extrapolation. Phil Trans R Soc Lond B.

[b9] Culman SW, Gauch HG, Blackwood CB, Thies JE (2008). Analysis of T-RFLP data using analysis of variance and ordination methods: a comparative study. J Microbiol Methods.

[b10] Danovaro R, Luna GM, Dell'Anno A, Pietrangeli B (2006). Comparison of two fingerprinting techniques, terminal restriction fragment length polymorphism and automated ribosomal intergenic space analysis, for determination of bacterial diversity in aquatic environments. Appl Environ Microbiol.

[b11] Dunbar J, Takala S, Barns SM, Davis JA, Kuske CR (1999). Levels of bacterial community diversity in four arid soils compared by cultivation and 16S rRNA gene cloning. Appl Environ Microbiol.

[b12] Dunbar J, Ticknor LO, Kuske CR (2000). Assessment of microbial diversity in four southwestern United States soils by 16S rRNA gene terminal restriction fragment analysis. Appl Environ Microbiol.

[b13] Engebretson JL, Moyer CL (2003). Fidelity of select restriction endonucleases in determining microbial diversity by terminal-restriction fragment length polymorphism. Appl Environ Microbiol.

[b14] Hartmann M, Frey B, Kölliker R, Widmer F (2005). Semi-automated genetic analyses of soil microbial communities: comparison of T-RFLP and RISA based on descriptive and discriminative statistical approaches. J Microbiol Methods.

[b15] Huber JA, Mark Welch DB, Morrison HG, Huse SM, Neal PR, Butterfield DA, Sogin ML (2007). Microbial population structures in the deep marine biosphere. Science.

[b16] Hughes JB, Hellmann JJ, Ricketts TH, Bohannan BJM (2001). Counting the uncountable: statistical approaches to estimating microbial diversity. Appl Environ Microbiol.

[b17] Hughes Martiny JB, Bohannan BJM, Brown JH, Colwell RK, Fuhrman JA, Green JL (2006). Microbial biogeography: putting microorganisms on the map. Nat Rev Microbiol.

[b18] Inagaki F, Nunoura T, Nakagawa T, Teske A, Lever M, Lauer A (2006). Biogeographical distribution and diversity of microbes in methane hydrate-bearing deep marine sediments on the Pacific Ocean Margin. Pro Natl Acad Sci U S A.

[b19] Kaplan CW, Kitts CL (2003). Variation between observed and true terminal restriction fragment length is dependent on true TRF length and purine content. J Microbiol Methods.

[b20] Lipp JS, Morono Y, Inagaki F, Hinrichs K-U (2008). Significant contribution of *Archaea* to extant biomass in marine subsurface sediments. Nature.

[b21] Liu WT, Marsh TL, Cheng H, Forney LJ (1997). Characterization of microbial diversity by determining terminal restriction fragment length polymorphisms of genes encoding 16S rRNA. Appl Environ Microbiol.

[b22] Ludwig W, Strunk O, Westram R, Richter L, Meier H, Yadhukumar (2004). ARB: a software environment for sequence data. Nucleic Acids Res.

[b23] Ricke P, Kolb S, Braker G (2005). Application of a newly developed ARB software-integrated tool for in silico terminal restriction fragment length polymorphism analysis reveals the dominance of a novel pmoA cluster in forest soil. Appl Environ Microbiol.

[b24] Santelli C, Orcutt B, Banning E, Moyer C, Bach W, Staudigel H, Edwards K (2008). Abundance and diversity of microbial life in the ocean crust. Nature.

[b25] Schippers A, Neretin LN (2006). Quantification of microbial communities in near-surface and deeply buried marine sediments on the Peru continental margin using real-time PCR. Environ Microbiol.

[b26] Schloss P, Handelsman J (2005). Introducing DOTUR: a computer program for defining operational taxonomic units and estimating species richness. Appl Environ Microbiol.

[b27] Sogin ML, Morrison HG, Huber JA, Welch DM, Huse SM, Neal PR (2006). Microbial diversity in the deep sea and the underexplored ‘rare biosphere’. Proc Natl Acad Sci U S A.

[b28] Sørensen KB, Teske A (2006). Stratified communities of active archaea in deep marine subsurface sediments. Appl Environ Microbiol.

[b29] Youssef NH, Elshahed MS (2008). Species richness in soil bacterial communities: a proposed approach to overcome sample size bias. J Microbiol Methods.

